# Natural scopoletin isolated from rubiaceous plants: a precursor for the synthesis of benzoylscopoletin and its cytotoxicity

**DOI:** 10.7717/peerj.21233

**Published:** 2026-05-05

**Authors:** Thanunchai Jaengthong, Artitaya Thiengsusuk, Sudathip Chantorn, Keerati Tanruean, Sutthichat Kerdphon, Markus Bacher, Tiwtawat Napiroon

**Affiliations:** 1 Department of Biotechnology, Faculty of Science and Technology, Thammasat University, Pathum Thani, Thailand; 2 Phytochemistry and Natural Products Laboratory, Department of Biotechnology, Faculty of Science and Technology, Thammasat University, Pathum Thani, Thailand; 3 Drug Discovery and Development Center, Office of Advanced Science and Technology, Thammasat University, Pathum Thani, Thailand; 4 Department of Biology, Faculty of Science and Technology, Pibulsongkram Rajabhat University, Phitsanulok, Thailand; 5 Department of Chemistry, Faculty of Science, Naresuan University, Phitsanulok, Thailand; 6 Institute of Chemistry of Renewable Resources, University of Natural Resources and Life Sciences (BOKU), Tulln/Donau, Austria

**Keywords:** Isolation, Synthesis, Scopoletin, Rubiaceae, Cytotoxicity

## Abstract

**Background:**

Scopoletin, a compound abundant in Rubiaceae such as *Lasianthus lucidus* and *Morinda citrifolia*, has demonstrated various activities, including antimicrobial activity, antinociceptive properties or pain relief and effects on cancer cell lines. Accordingly, it has significant potential as a precursor for synthetic bioactive agents for reducing pain such its derivative includes benzene ring. However, very little has been reported regarding normal cellular effects or human skin irritation of such synthetic compounds, knowledge which is necessary if they are to be further developed. Thus, the aim of this study was to isolate scopoletin from Thai Rubiaceae, synthesize a derivative, and test its cytotoxicity.

**Materials and Methods:**

Stem bark of *L. lucidus* and fruits of *M. citrifolia* were extracted and the extract partitioned into hydrophilic and lipophilic fractions. Lipophilic extracts were further fractionated by column chromatography and analysed by High-Performance Liquid Chromatography with Diode Array Detection (HPLC-DAD). Purified scopoletin was used for the synthesis of its benzoate derivative and their structures were confirmed with the help of Nuclear Magnetic Resonance (NMR) spectroscopy. The cytotoxicity of the derivative compound was tested in the immortalized human keratinocyte HaCaT cell line.

**Results:**

The lipophilic extracts displayed scopoletin contents of 16.14% and 13.94%, respectively. Scopoletin was completely converted to a target derivative, 6-methoxy-2-oxo-4a,8a-dihydro-2H-chromen-7-yl benzoate. This compound exhibited an IC_50_ value of 182.95 ± 6.15 µg/mL, with cell viability increased at concentrations less than 15.63 µg/mL but decreased at concentrations of 31.25 to 500 µg/mL.

**Conclusions:**

We completely actualized both isolation and synthesis to achieve a high yield of the target scopoletin and benzoylscopoletin. The cytotoxicity evidence obtained from this study supports the establishment of safe dosage limits, which have not been previously reported. This finding provides a scientific basis for further drug development. The analyses demonstrate reproducible isolation of scopoletin and synthesis of the derivative.

## Introduction

Rubiaceous plants are important sources of secondary metabolites with a broad diversity of chemical structures and biological activities that promise great potential in the production of bioactive compounds (Martins & Nunez, 2015). Rubiaceae have gained attention from the medicinal perspective and drug discovery research in finding bioactive compounds (Pereira & Meireles, 2010). Many plants in this family have widespread usage in traditional medicine and conspicuously demonstrate anti-inflammatory, analgesic, antimicrobial, and antioxidant activities, along with effects on vascular diseases and the central nervous system (Heitzman et al., 2005). Of particular interest is scopoletin, a coumarin derivative widely prevalent with high content in Rubiaceae genera, including *Alibertia, Augusta, Fadogia, Galianthe, Hymenodictyon, Lasianthus, Morinda, Ophiorrhiza, Plocama, Psychotria, Sabicea*, and *Saprosma* (Martins & Nunez, 2015). Interestingly, reports highlight *Lasianthus* and *Morinda* as containing especially high levels of scopoletin (Napiroon et al., 2018; Jia, Lan & Wu, 2020) in leaves, bark, and fruits. For example, fruit and leaf juice from *Morinda citrifolia*, commonly known as “noni” in the Asia-Pacific region, contain scopoletin, and *M. citrifolia* is used for the treatment of diabetes, regulation of blood pressure, a poultice on wounds, hypertension, and stimulation of appetite (Peter & Peter, 2018; Souza et al., 2018; McClatchey, 2002). Moreover, the European Commission approved noni fruit juice as a novel and safe health food in Europe (Official Journal of the European Union, 2003). Similarly, several *Lasianthus* species such as *L. lucidus* and *L. stipularis* are used in traditional medicine for wound treatment and to reduce fevers caused by infections (Rai & Lalramnghinglova, 2011; Napiroon et al., 2018, 2021) these species are likewise reported to be rich in scopoletin.

Scopoletin is a phenolic coumarin also known as 6-methoxy-7-hydroxycoumarin. It is commonly found in many medicinal and edible plants that play important roles in human health (Gnonlonfin, Sanni & Brimer, 2012) and has been utilized in traditional medicine formulations in Africa, Asia, and Europe (Xia et al., 2007). Naturally, scopoletin is often involved in plant defence against microbial infection (Gnonlonfin, Sanni & Brimer, 2012). *In vitro*, scopoletin can stabilize radicals *via* a hydrogen atom transfer mechanism, indicative of having antioxidant activity (Antika et al., 2022). *In vivo*, oral administration of 10 mg/kg scopoletin to hypertensive rats has been reported to inhibit NO production, further supporting its antioxidant property (Chen, Pittman & Popel, 2008; Armenia et al., 2019). Additionally, Cai et al. (2013) demonstrated scopoletin derivatives to have anti-angiogenic activity in human cancer cell lines. Such derivatives are also promising candidates for quenching free radicals (Jamuna et al., 2015). These findings showing multiple bioactivities of scopoletin derivatives highlight a need for future studies exploring the functional potential of new scopoletin derivatives.

## Materials and Methods

### Plant identification and collection

Two rubiaceous plants, *Lasianthus lucidus* and *Morinda citrifolia*, were selected as the main materials for scopoletin isolation. Stem bark of *L. lucidus* was collected during the fruiting stage, between November and December 2024. Fruit of *M. citrifolia* was collected from agricultural areas in October 2024. All voucher specimens of *L. lucidus* (collector no. NT101) and *M. citrifolia* (collector no. NT103) were deposited at the Department of Biotechnology, Faculty of Science and Technology, Thammasat University and the Bangkok Forest Herbarium. Field collection for this study did not require a specific permit because the plant materials were obtained from agricultural areas and disturbed areas with the owner’s permission. The species are not protected or endangered. Field surveys and specimen collections were conducted in public lands and degraded agricultural areas outside of protected conservation zones. This work was carried out under the research project of Thammasat University (Project Code: TUFF26/2568). Fertile and vegetative organs were compared with type specimens of each species at the K and L herbaria, using photographs available on the web and related literature (Zhu, Roos & Ridsdale, 2012; Gardner, Sidisunthorn & Chayamarit, 2015). The fertile and vegetative organs of plant samples were observed under a stereo microscope for identification. Herbarium acronyms follow the Index Herbariorum (The New York Botanical Garden, 2026).

### Extraction and isolation

*Lasianthus lucidus* stem bark was chopped and dried in an oven at 40–45 °C over seven to 10 days. *Morinda citrifolia* fruits were also chopped and dried in an oven at 45–50 °C for the same time. Each dried materials were powdered *via* an electronic mill. For each powdered sample, 300 g dry weight was macerated with methanol (CH_3_OH) for 7 days in a dark cabinet at room temperature. Afterwards, the liquid phase was filtered through filter article (Whatman No. 1) and the solvent evaporated by a rotary evaporator at 45 °C until the extract reached a viscous stage. Then, the concentrated extracts were partitioned into two phases in separating funnels, comprising a hydrophilic phase in distilled water and a lipophilic phase in chloroform. Only the lipophilic phase was evaporated and stored in an ember glass bottle at 0–4 °C for subsequent experiments.

**Figure 1:**
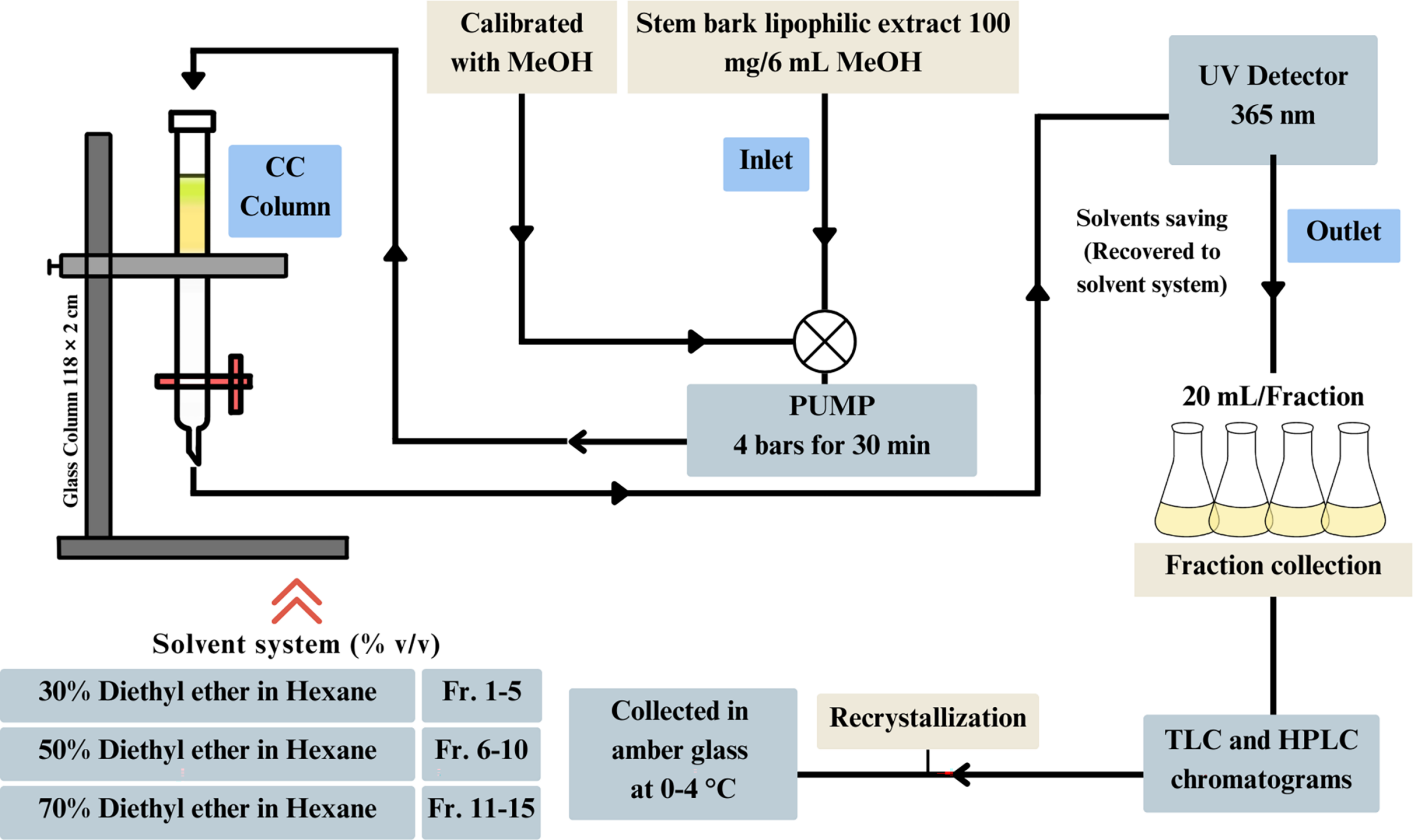
The isolation process of natural scopoletin from rubiaceous plants.

For the isolation of scopoletin, the lipophilic extract was fractionated using column chromatography together with medium pressure. A total of 100 mg extract (dry weight) was dissolved in 6 mL MeOH and then loaded onto a glass column (118 × 2 cm), filled with silica gel as stationary phase (50 g, 40 to 63 µm particle size; Supelco, Sigma-Aldrich, Burlington, MA, USA). Fractions of 20 ml were collected with a gradient solvent system comprising five fractions each of 30% diethyl ether in hexane (fractions 1 to 5), 50% diethyl ether in hexane (fractions 6 to 10), and 70% diethyl ether in hexane (fractions 11 to 15). Before and after each run, the column was washed and calibrated with methanol (analytic grade, Merck, Rahway, NJ, USA) for 30 min with the pressure set to four bars. All fractions were checked for purity with thin layer chromatography (TLC) and UV detection (wavelength 365 nm). Fractions with similar composition were combined and recrystallized with diethyl ether before being collected in ember glass at 0–4 °C for future bioassays. Our process designing is illustrated in Fig. 1.

### Chromatographic analysis

For TLC, the lipophilic extracts were placed on precoated silica gel 60 F254 plates (20 × 20 cm; Merck, Rahway, NJ, USA) using a solvent system of hexane:ethyl acetate (7:3 v/v) and detected under UV irradiation (365 and 254 nm). The R_f_ value of each fluorescent spot was determined as a TLC pattern. For high-performance liquid chromatography (HPLC), 1 mg/mL of lipophilic extracts or pure compounds in methanol (CH_3_OH; HPLC grade, Merck, Rahway, NJ, USA) were prepared and filtered through a 0.45 µm nylon filter. For HPLC analyses a solvent system comprising 60% v/v methanol, gradient 60% to 100% (HPLC grade; Merck, Rahway, NJ, USA), in 40% v/v aqueous buffer (0.015 M orthophosphoric acid pH 3 and 0.015M tetrabutylammonium hydroxide) was used, which has previously been demonstrated suitable as a mobile phase for coumarin detection (Vajrodaya, 1998; Vajrodaya et al., 1998). In addition, HPLC with a solvent system consisting of 0.5% acetic acid in methanol (HPLC grade; Merck, Rahway, NJ, USA), gradient 0% to 100%, was used to confirm the single peaks of pure compounds. HPLC was performed on an Agilent 1100 series instrument with detection by a UV photodiode arrays detector (DAD) at wavelengths of 230, 254, and 280 nm.

### Synthesis preparation

Synthesis equipment and reagents were as follows: column chromatography, silica gel 60 spherical, 38–63 µm, Wakosil^®^; TLC, silica gel 60 aluminium plate, F254, 0.20 mm, Macherey-Nagel GmbH & Co. KG, Düren, Germany. The cap tube was heat-tolerant to 180 °C (glass DWK DURAN^®^, Baden-Württemberg, Germany). HPLC-DAD was performed on an Agilent 1100 series with a diode array detector (Agilent, Santa Clara, CA, USA), Nuclear Magnetic Resonance Spectroscopy AVANCE III HD 300 MHz (Liquid) (Bruker, Fällanden, Switzerland).

**Figure 2:**
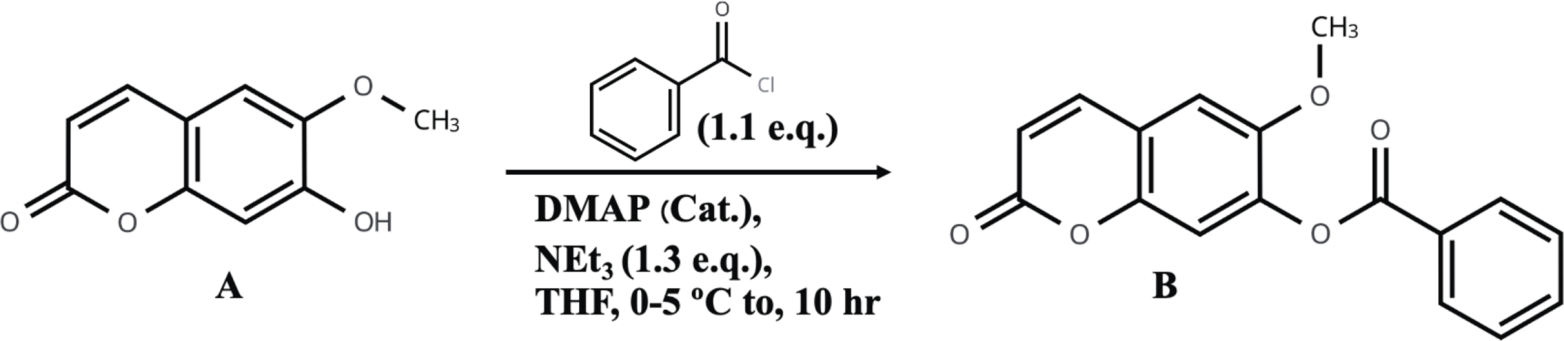
The scheme outlines how synthesis from natural scopoletin of rubiaceous plants.

In the first step of synthesis, 0.07 g (0.36 mmol) scopoletin, 0.0479 g triethylamine, 4-dimethylaminopyridine (DMAP, catalytic amount) and 1 mL tetrahydrofuran (THF) were combined in a cap tube, then stirred with a magnetic bar in a cooling water bath (0–5 °C). Secondly, 0.0563 g benzoyl chloride in 0.2 mL THF was added, then incubated at 0–5 °C with stirring for 10 h. The scheme of synthesis is illustrated in Fig. 2. After the incubation, the compound was dried by evaporation and redissolved in 10 mL hexane:ethyl acetate (8:2% v/v). The liquid solution was purified by column chromatography and eluted with 100 mL of an isocratic system (hexane:ethyl acetate, 8:2% v/v). A fraction volume of 50 mL was collected, and fractions that demonstrated similar composition on TLC and HPLC-DAD at 365 nm were combined. Then, the solvent in fraction was subsequently evaporated and added ethyl acetate (AR grade) to the product, followed by a second evaporation step to remove it, yielding the final product powder. Finally, the powdered material was recrystallized in diethyl ether, NMR structure elucidation was performed in comparison with scopoletin, and the synthesized product was identified according to IUPAC nomenclature.

### MTT bioassay

The thiazolyl blue tetrazolium bromide (MTT) bioassay was used to determine compound impact on cell viability and its cytotoxicity. MTT dissolves to form a yellowish solution in phosphate buffered saline and is reduced to an insoluble purple crystal (formazan) by mitochondrial reductase enzymes in living cells. This assay was carried out according to established procedure with HaCaT as the cell model, an immortalized human keratinocyte line provided by Cell Lines Service GmbH (Eppelheim, Baden-Württemberg, Germany).

HaCaT cells were stored and cultured at the Drug Discovery and Development Center, Office of Advanced Science and Technology, Thammasat University, Thailand. HaCaT cells were seeded in a 96-well plate at a density of 1 × 10^4^ cells/well and cultured in Dulbecco’s Modified Eagle Medium (DMEM) containing 10% FBS and 1% antibiotic–antimycotic and incubated for overnight at 37 °C in a cell culture incubator with humidified atmosphere of 5% CO_2_. The cells were exposed to tested compounds for 48 h and cell viability was examined using the MTT assay. Briefly, 20 µL of 5 mg/mL of MTT in PBS was added to each well of the 96-well plate and the plate was incubated for 3 h in the cell culture incubator. Subsequently, the MTT solution was discarded and 100 µL of DMSO was added to each well to dissolve formazan crystals. The optical density (OD) was measured at 590 nm (with background subtraction using blank wells) using SPECTROstar Nano microplate reader. All experiments were conducted using cells within a passage range of 15–25. Our experiment tested the final pure compound at concentrations of 3.91, 7.81, 15.63, 31.25, 62.50, 125, 250, and 500 μg/mL, and included untreated controls. The untreated control utilized medium without pure compound which representing 100% viability. The half-maximal inhibitory concentration (IC_50_) was calculated using the CalcuSyn program based on the median-effect principle, utilizing both linear and logarithmic transformations to establish the dose-response correlation. Each experiment was conducted in triplicate, and all concentrations were tested with replicates. This experimental design aimed to evaluate the trend of morphological changes and cytotoxicity in the immortalized human keratinocyte HaCaT cell line.

## Results

### Determination of scopoletin in extracts

**Figure 3:**
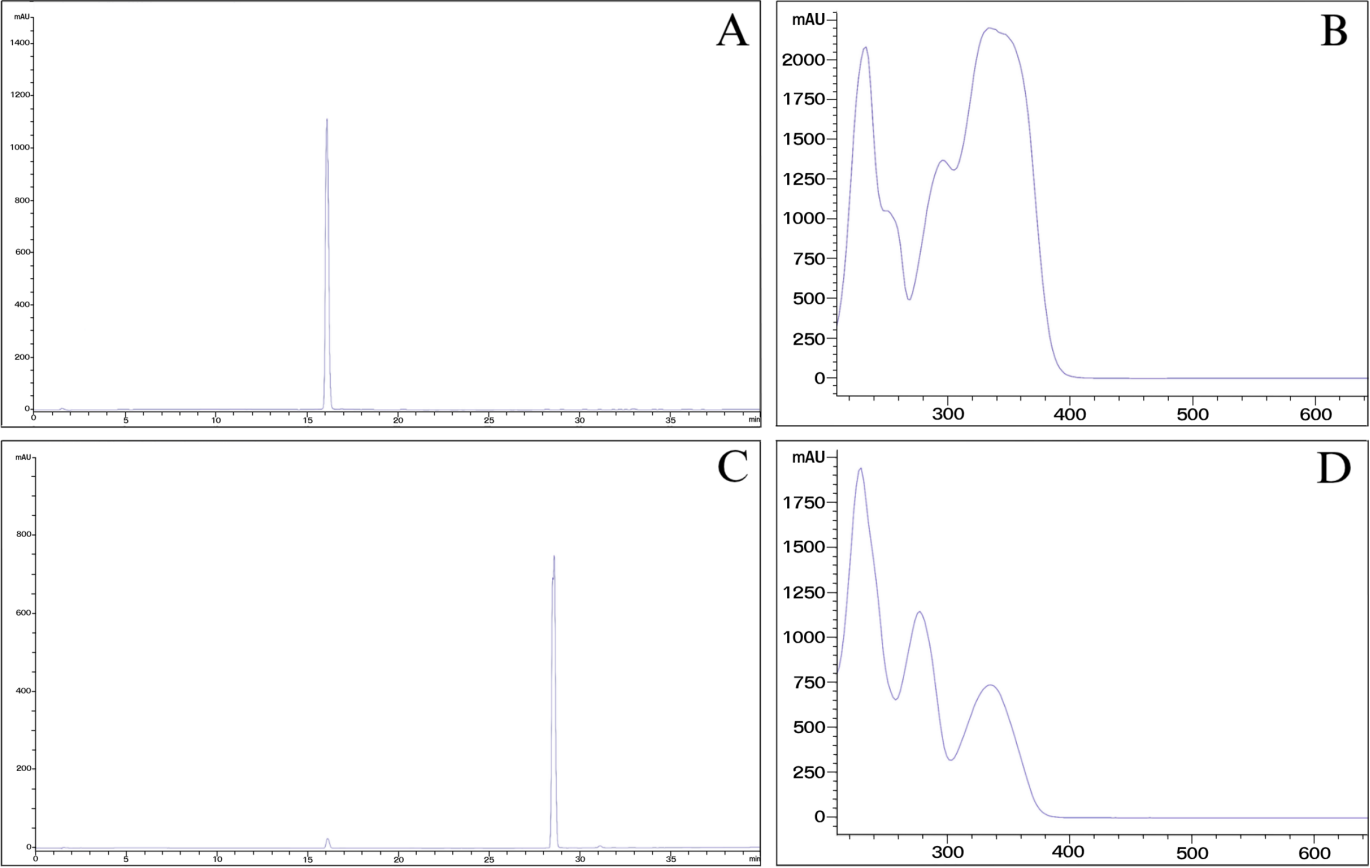
Comparative HPLC-DAD chromatograms of scopoletin and derivative compound.

Starting from 500 mg of each lipophilic extract, the fractionation of *Lasianthus lucidus* stem bark and *Morinda citrifolia* fruit provided yellowish crystalline powders of 80.71 mg (16.14% yield) and 69.72 mg (13.94% yield), respectively. Fractions that exhibited similar TLC chromatograms, specifically fractions No. 8 to No. 13, were combined and further purification over sephadex was carried out. Evaporation of this combination and then eluted by sephadex column chromatography with methanol isocratic elution. The TLC chromatogram of each fraction was investigated for the presence of scopoletin based on comparison with the retention time and UV spectrum of standard compound (Sigma-Aldrich^®^, Burlington, MA, USA). The yellowish crystalline material that appeared at the bottom of the flask was recrystallized in diethyl ether (≥99.9% purity, analytical grade, Merck, Rahway, NJ, USA). The HPLC profile showed characteristics of scopoletin at the retention time of 16.148 min (Fig. 3). The corresponding NMR chemical shifts in CDCl_3_ are listed in Table 1 and are in agreement with those previously reported for scopoletin (Darmawan et al., 2012). The NMR spectra are shown in Supplemental 1A and 1B.

**Table 1:** NMR data of scopoletin and new derivative (^1^H, 300 MHz and ^13^C in CDCl_3_).

Position	Scopoletin		New derivative	
	δ_H_ (mult., *J* Hz)	δ_C_	δ_H_ (mult., *J* Hz)	δ_C_
1	–	161.46	–	160.78
2	6.20 (d, ^1^H, 9.5)	113.41	6.43 (d, ^1^H, 9.5)	116.64
3	7.53 (d, ^1^H, 9.5)	143.31	7.69 (d, ^1^H, 9.5)	142.98
4	–	111.50	–	117.08
5	7.19 (s, ^1^H)	110.13	7.02 (s, ^1^H)	109.62
6	–	144.02	–	148.53
7	–	150.26	–	143.41
8	6.77 (s, ^1^H)	103.19	7.22 (s, ^1^H)	112.36
9	-	149.71	-	148.82
10	3.88 (s, ^1^H)	56.42	3.68 (s, ^1^H)	56.60
11			–	164.30
12			–	134.08
13			8.26–8.17 (m, ^1^H)	130.57
14			7.56–7.51 (m, ^1^H)	128.81
15			7.68–7.65 (m, ^1^H)	128.78
16			7.56–7.51 (m, ^1^H)	128.81
17			8.26–8.17 (m, ^1^H)	130.57

### Synthesis and purification of a new scopoletin derivative

The new derivative compound was completely synthesized as pure compound. Briefly, scopoletin purified from fractionation was combined with DMAP and THF, then stirred in a cooling water bath. Next, benzoyl chloride in THF was added and the resultant solution stirred for 10 h. Foaming with ethyl acetate (AR grade) under vacuum (0.08–0.04 MPa) was then performed to yield 138.65 mg of white powder, approximately two times the mass when compared with the precursor. After washing powder with methanol (analytical grade), 69.32 mg of the product was obtained, representing a 99% yield. The HPLC chromatogram of the product revealed a single peak at 28.616 min.

**Figure 4:**
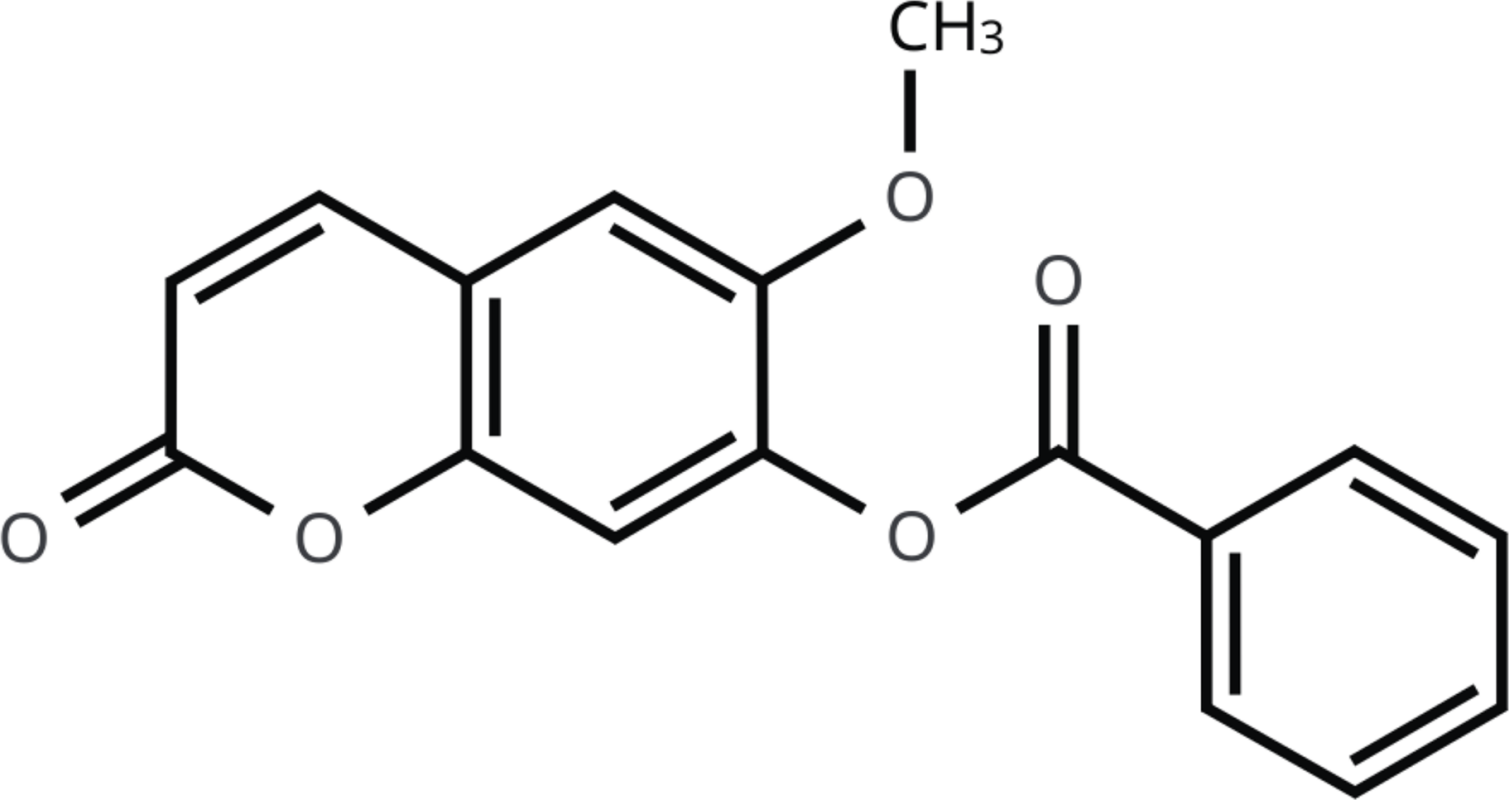
The chemical structure of derivative compound.

NMR structure elucidation of the new derivative clearly showed it to differ from scopoletin. Specifically, the ^1^H NMR (300 MHz, CDCl_3_): *δ* 8.26–8.15 (m, 2H), 7.76–7.60 (m, 2H), 7.59–7.48 (m, 2H), 7.22 (s, 1H), 7.02 (s, 1H), 6.43 (d, *J* = 9.6 Hz, 1H), 3.86 (s, 3H). ^13^C NMR (75 MHz, DMSO-*d*_*6*_): *δ* 164.8, 160.8, 148.8, 148.5, 143.3, 1,423.0, 134.1, 130.6, 128.8, 117.1, 116.6, 112.4, 109.6, 56.6. On Fourier transform infrared spectroscopy (KBr, *ν*), peaks were observed at 3,053, 2,973, 2,938, 2,918, 1,728, 1,598, 1,566, 1,499, 1,463, 1,449, 1,419, 1,385, 1,352, 1,266, 1,235, 1,198, 1,169, 1,130, 1,096, 1,074, 1,053, 1,018, and 1,003 cm^−1^. According to IUPAC nomenclature, the compound is 6-methoxy-2-oxo-4a,8a-dihydro-2H-chromen-7-yl benzoate. Its chemical structure is depicted in Fig. 4 and NMR spectra are shown in Supplemental 2A and 2B.

### MTT bioassay experiments

**Figure 5:**
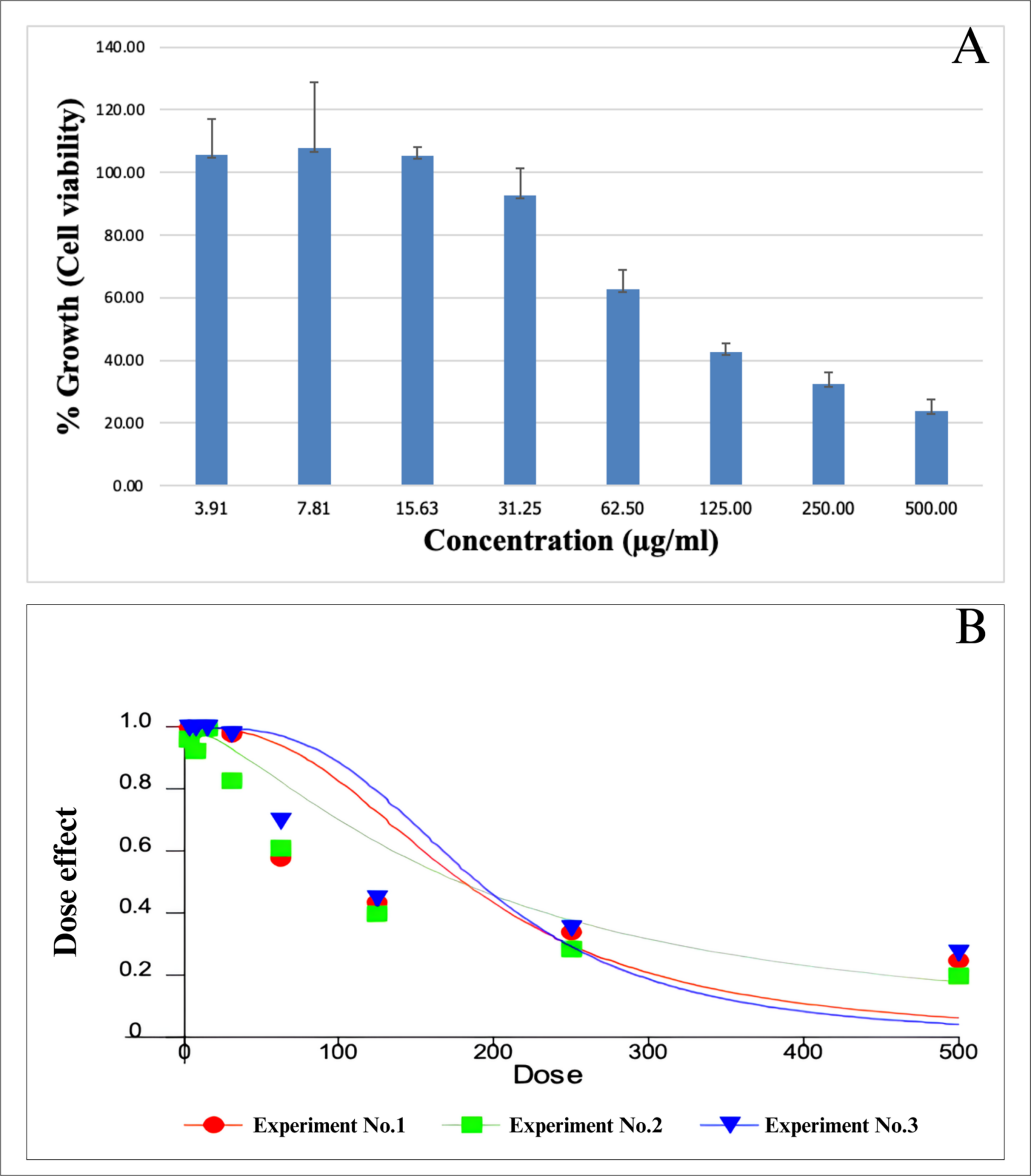
Cell viability displayed as the percentage of new derivative compound and control (means ± SD), triplicate independent experiments.

**Figure 6:**
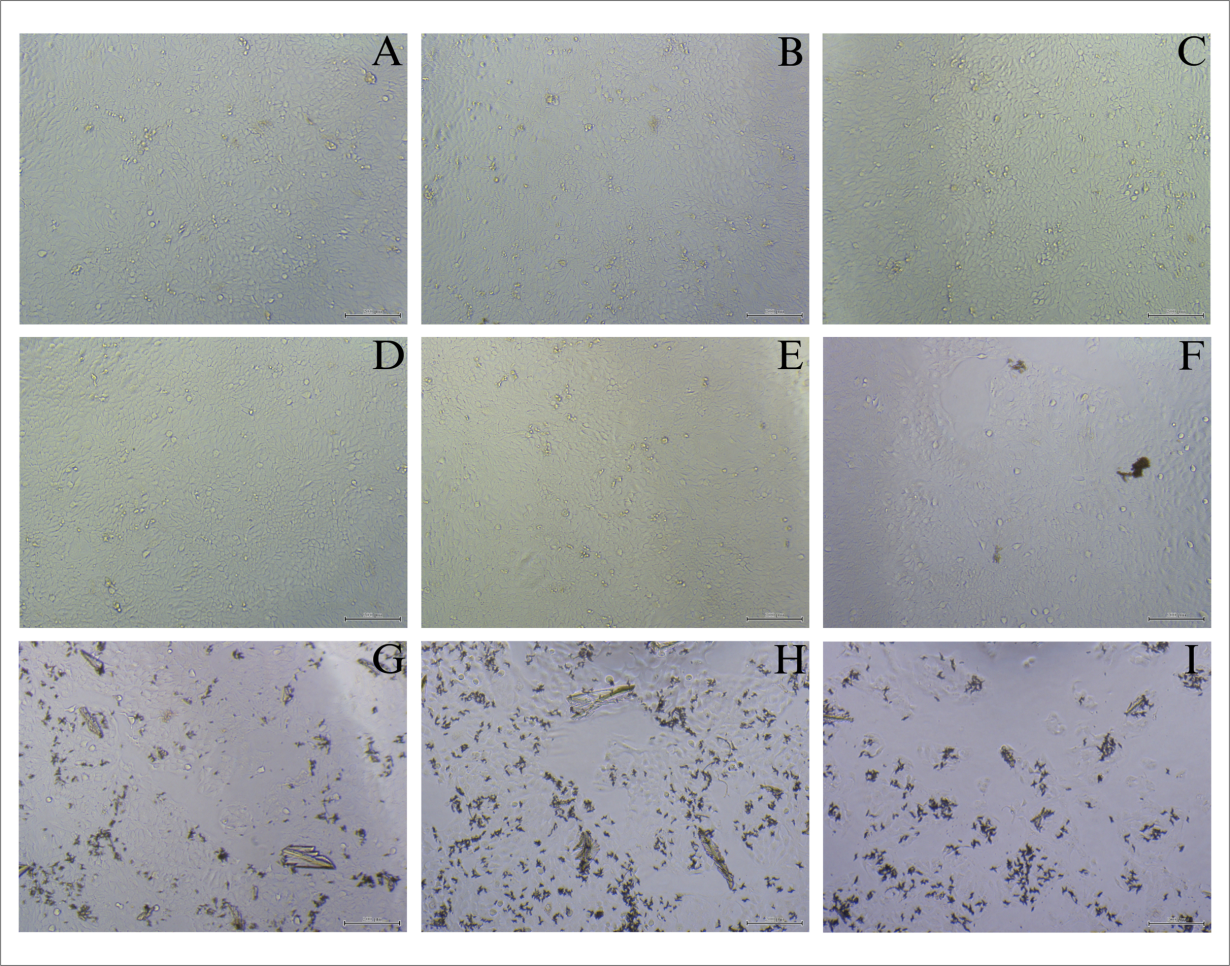
Morphological characteristics of the immortalized human keratinocyte HaCaT cell line after treatment at 48 h in different concentration. (A) Control, (B) 3.91 µg/mL, (C) 7.81 µg/mL, (D) 15.63 µg/mL, (E) 31.25 µg/mL, (F) 62.50 µg/mL, (G) 125 µg/mL, (H) 250 µg/mL and (I) 500 µg/mL.

After 48 h, immortalized human keratinocyte HaCaT cells treated with scopoletin benzoate exhibited increased viability; specifically, concentrations of 3.91, 7.81, and 15.63 µg/mL resulted in respective percentage values of 105.75 ± 11.40, 107.70 ± 21.13, and 105.25 ± 2.72. However, treatment with higher concentrations resulted in decreased viability, with 31.25, 62.50, 125, 250, and 500 µg/mL yielding respective percentage values of 92.70 ± 8.69, 62.88 ± 6.11, 42.69 ± 2.61, 32.58 ± 3.54, and 23.93 ± 3.73 (Fig. 5). The reduction in cell viability associated with increased concentration is in agreement with the dose-effect relationship illustrated in the CalcuSyn plot. An IC_50_ value of 182.95 ± 6.15 µg/mL was calculated using the CalcuSyn program. Representative images showing the morphological characteristics of cells with increased viability are shown in Figs. 6B–6D, while images of cells with reduced viability are presented in Figs. 6E–6I. These results suggest that the scopoletin derivative may be considered non-toxic at concentrations below the IC_50_, but significantly impairs cell viability at higher concentrations which may be partially attributed to the increased final concentration of DMSO (up to 1%) required for compound solubility at these levels.

## Discussion

### Scopoletin isolation and derivative synthesis

This study carried out column chromatography to isolate scopoletin from lipophilic extracts of *Lasianthus lucidus* stem bark and *Morinda citrifolia* fruits. Column chromatography is an effective method for isolating compounds based on their different adsorption to a stationary phase and elution with a varying polarity mobile phase (Srivastava et al., 2021). The raw plant materials were selected based on previous literature reviews, which found extracts of both plant species to yield high quantities of scopoletin. This is particularly supported by reports from Thailand and Southeast Asia, which serves as a natural habitat for these plants wherein they can also be found in agricultural areas (Napiroon et al., 2018; Hou et al., 2025). The isolated compound appeared as a yellowish crystal and was further investigated by TLC using the mobile phase hexane:ethyl acetate (6:4 and 7:3 v/v). TLC serves as an excellent first-pass, low-cost screening tool to quickly narrow down possible identifications. Our solvent system developed for scopoletin detection in plant extracts is similar to others used in the literature (Darmawan et al., 2012; Napiroon et al., 2018). In addition to chromatography, HPLC-DAD and HPLC-MS analysis are often performed to ensure precise determinations. HPLC can then be used for more detailed and accurate qualitative and/or quantitative fast screening. In the present study, chromatography of scopoletin with methanol:buffer (60:40% v/v) resulted in a single peak at 3.34 min, whereas chromatography with 0.5% acetic acid in methanol (0–100% v/v) resulted in a single peak at 16.14 min. Both results support the purity of the scopoletin isolated and recrystallized from extracts. The mobile phase system employed in this work was designed to analyse scopoletin from both acid-containing and non-acid systems. Examples of mobile phase systems commonly used for scopoletin analysis that have been demonstrated to offer relatively high accuracy include methanol:aqueous buffer or aqueous (Napiroon et al., 2018; Buathong et al., 2019; Patel et al., 2021; Chen et al., 2024), formic acid:acetonitrile (Tasfiyati et al., 2023; Sam-ang et al., 2023), and glacial acetic acid:methanol (Shinde et al., 2014; Nahata et al., 2018) systems.

The isolated compound was identified by characterization of its chemical structure based on ^1^H and ^13^C NMR spectral data. The ^1^H-NMR spectrum showed four aromatic protons (δH 6.20, 6.77, 7.19, and 7.53 ppm) and one methoxy group (δH 3.88 ppm), while the ^13^C-NMR spectrum indicated ten carbons. In the synthesized scopoletin derivative, the OH group at carbon position C−7 was completely replaced by a benzoyl moiety, which was unequivocally proved by the presence of additional aromatic signals in both the ^1^H and ^13^C NMR spectra. Our synthesis approach aligns with the development of scopoletin derivatives by Cai et al. (2013), wherein successful process and synthesis were also executed at the C−7 position of scopoletin.

### Toxicity investigation

The present work represents a preliminary investigation into the effects of a novel synthetic scopoletin on HaCaT cells, an immortalized human keratinocyte cell line commonly utilized for viability assays in studies of immunological and inflammatory responses (Colombo et al., 2017). We determined that at low concentrations (3.91, 7.81, and 15.63 µg/mL), the synthetic scopoletin derivative does not exert cytotoxic effects but rather notably enhances the proliferation of HaCaT cells. However, higher concentrations were observed to negatively affect HaCaT cell viability. Antika et al. (2022) previously reported scopoletin itself to possess antimicrobial, anti-inflammatory, and immunomodulatory properties that are mediated in part through suppression of cytokine production, including TNF-α, IL-1β, and IL-6. A previous study by Seo et al. (2024) likewise investigated the structurally related compound isoscopoletin for its anti-inflammatory effects on HaCaT cells; the findings suggested that it has potential for modulating skin inflammation. In Cai et al. (2013). reported the biological evaluation of 25 scopoletin derivatives, ultimately identifying the three compounds 7-[2-(4-bromo-phenyl)-2-oxo-ethoxyl]-6-methoxy-2*H*-chromen-2-one, 7-[2-(4-chloro-phenyl)-2-oxo-ethoxyl]-6-methoxy-2*H*-chromen-2-one, and 7-(2-hydroxy-3(piperidin-1-yl)-propoxy)-6-methoxy-2*H*-chromen-2-one exhibiting significant anti-angiogenesis activities against human cancer cell lines. These same derivatives also inhibited angiogenesis in a chicken embryo *in vivo* model. Interestingly, structural modifications of scopoletin in which the hydroxyl (OH) position is replaced with some heteroatom instead of carbon or a halogen, similar to our derivative compound, usually promote bioactivity. In an *Artemia in vitro* model, a scopoletin derivative including a benzene ring and chorine slightly showed less toxicity at 12 h than did the unsubstituted scopoletin (Liu et al., 2023). In addition, other synthetic compounds were shown to have a bioactivity enhancing effect and control, which are advantageous as they can be further utilized for pharmaceutical agents (China et al., 2020; Rusu et al., 2023).

Halogens and compounds containing a heterocyclic ring are often crucial as reactive agents in chemical synthesis processes, especially in pharmaceutical production, as the resultant products often exhibit good pharmacological efficacy (Robin & Rousseau, 2002; China et al., 2020). However, compounds incorporating these substances are also often reported to cause skin and mucous membrane irritation. Accordingly, synthetic compounds that have been developed for practical use typically undergo thorough testing and their usage amounts are controlled to ensure safety and prevent harmful side effects for users. In addition, its cytotoxicity may be attributed to the appearance of the benzoyl moiety, which often increases the lipophilicity. These findings align with those of Cao et al. (2025), highlighting that the benzoyl moiety in molecule plays a crucial role in facilitating effective cellular entry, which is highly beneficial for therapeutic and pharmacological purposes.

Several simple coumarin structures that have been designed as new derivatives, including derivatives of coumarin and scopoletin, have good potential for antimicrobial activity against *Mycobacterium* (Reddy, Kongot & Kumar, 2021) and various human cancer cell lines (Cai et al., 2013). From *in vitro* assays, coumarins and derivatives have mostly shown potential as antimicrobial agents, low-toxicity antibiotics, active pharmacophores, and for their effects on cancer cells (Reddy, Kongot & Kumar, 2021). However, the effects of these derivatives on normal human cells have not yet been reported. This work is a first report of a new scopoletin derivative that appears to contain some heteroatom instead of carbon and its demonstrated effects on the immortalized human keratinocyte HaCaT cell line, for which the IC_50_ value was 182.95 ± 6.15 µg/mL. If the inhibitory concentrations of coumarin derivatives against microbials and cancer cells remain low, such a relatively high IC_50_ value indicates a relatively high likelihood that the compound is safe and non-toxic. Therefore, future studies should further characterize the mechanisms of action to confirm the potential biological activities of this compound and support its development as a therapeutic agent.

## Conclusions

Lipophilic extracts of *Lasianthus lucidus* stem bark and *Morinda citrifolia* fruit produced scopoletin yields of 16.14% and 13.94%, respectively. A derivative synthesized from this scopoletin, 6-methoxy-2-oxo-4a,8a-dihydro-2H-chromen-7-yl benzoate, demonstrated a significant effect on the immortalized human keratinocyte HaCaT cell line, with morphological changes observed after treatment. The IC_50_ value was 182.95 µg/mL, above which a significant reduction in cell viability was observed at 48 h. At lower concentrations, the compound is expected to be non-toxic in normal cells, especially human skin cells. To our knowledge, this constitutes the first study to evaluate the effects of this compound in the specified cell line. The chemical structure of this derivative tends to align with some antimicrobial agents; however, further studies characterising its biological activities will be needed in the future. This unique compound may have promise as an efficient synthetic compound with potential for future advances.

## Supplemental Information

10.7717/peerj.21233/supp-1 Supplemental Information 1 1H NMR spectra of scopoletin.

10.7717/peerj.21233/supp-2 Supplemental Information 2 13C NMR spectra of scopoletin.

10.7717/peerj.21233/supp-3 Supplemental Information 3 1H NMR spectra of new derivative compound.

10.7717/peerj.21233/supp-4 Supplemental Information 4 13C NMR spectra of new derivative compound.

10.7717/peerj.21233/supp-5 Supplemental Information 5 Cytotoxicity data experiments raw data.

## Additional Information and Declarations

### Field Study Permissions

The following information was supplied relating to field study approvals (*i.e*., approving body and any reference numbers):

Yes. The plant materials were collected from public agricultural areas with the assistance of local individuals, as detailed in the confidential information section.

### Data Availability

The following information was supplied regarding data availability:

The NMR spectra are available in the Supplemental Files.
