# Supplementary material for: Natural scopoletin isolated from rubiaceous plants: a precursor for the synthesis of benzoylscopoletin and its cytotoxicity
Source: PeerJ. 2026 May 5;14:e21233. doi: 10.7717/peerj.21233 (PMC13155236; doi:10.7717/peerj.21233)
Supplement: Supplemental Information 1 [file peerj-14-21233-s001.pdf]

Center of Scientific Equipment for Advanced Research
Thammasat University

NMR: Ascend TM 600/
Avance III HD
Bruker, Switzerland

Current Data Parameters

NAME	NA2-680838_SC-H-NMR
EXPNO	1
PROCNO	1

F2 - Acquisition Parameters

Date_	20250707
Time	15.38 h
INSTRUM	spect
PROBHD	Z114607_0275 (
PULPROG	zg30
TD	65536
SOLVENT	CDCl3
NS	64
DS	2
SWH	9615.385 Hz
FIDRES	0.293438 Hz
AQ	3.4078720 sec
RG	119.43
DW	52.000 usec
DE	6.50 usec
TE	298.0 K
D1	1.50000000 sec
TD0	1
SFO1	600.1336008 MHz
NUC1	1H
P0	3.33 usec
P1	10.00 usec
PLW1	25.04999924 W

F2 - Processing parameters

SI	65536
SF	600.1300555 MHz
WDW	EM
SSB	0
LB	0.30 Hz
GB	0
PC	1.00

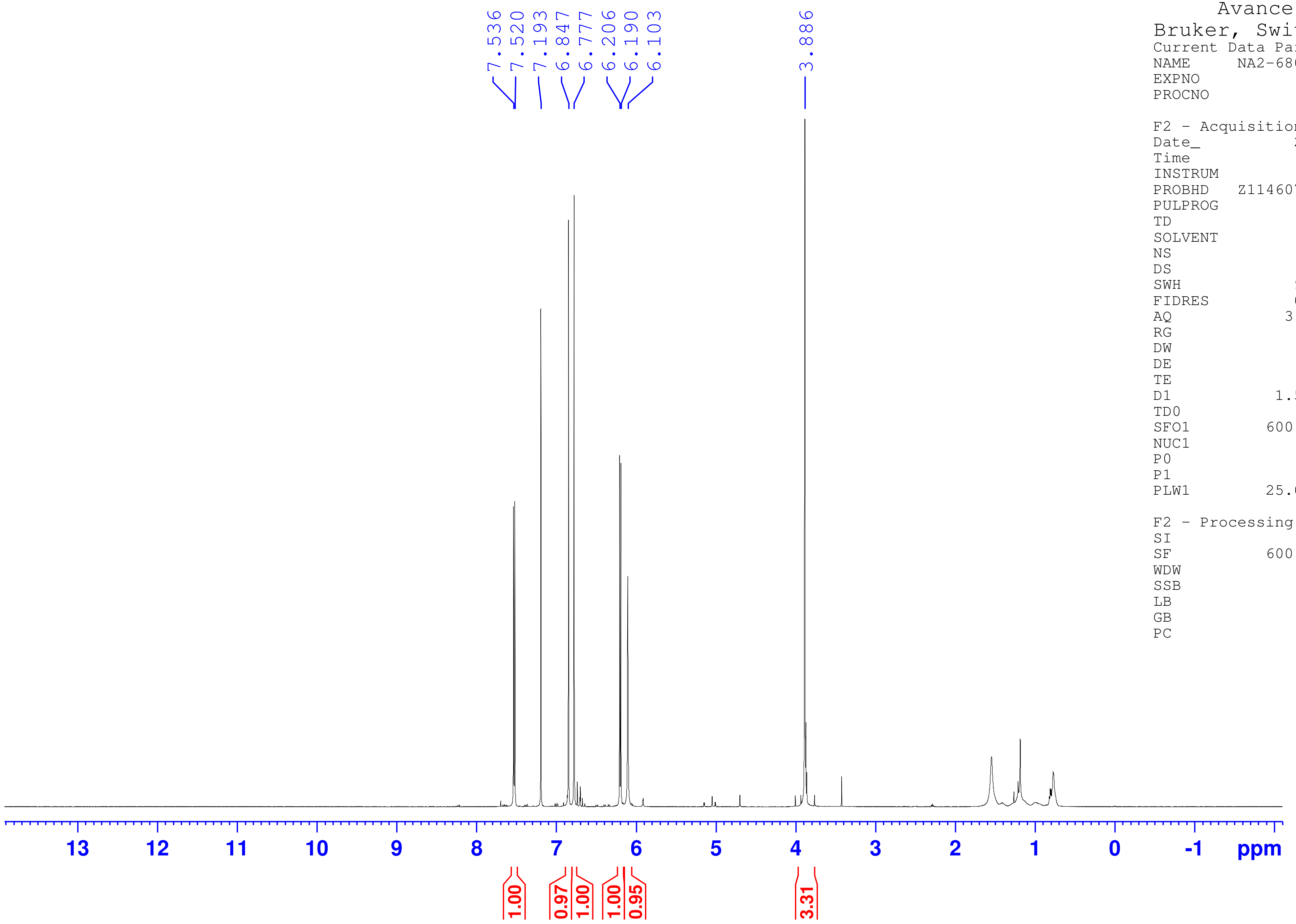

Center of Scientific Equipment for Advanced Research
Thammasat University

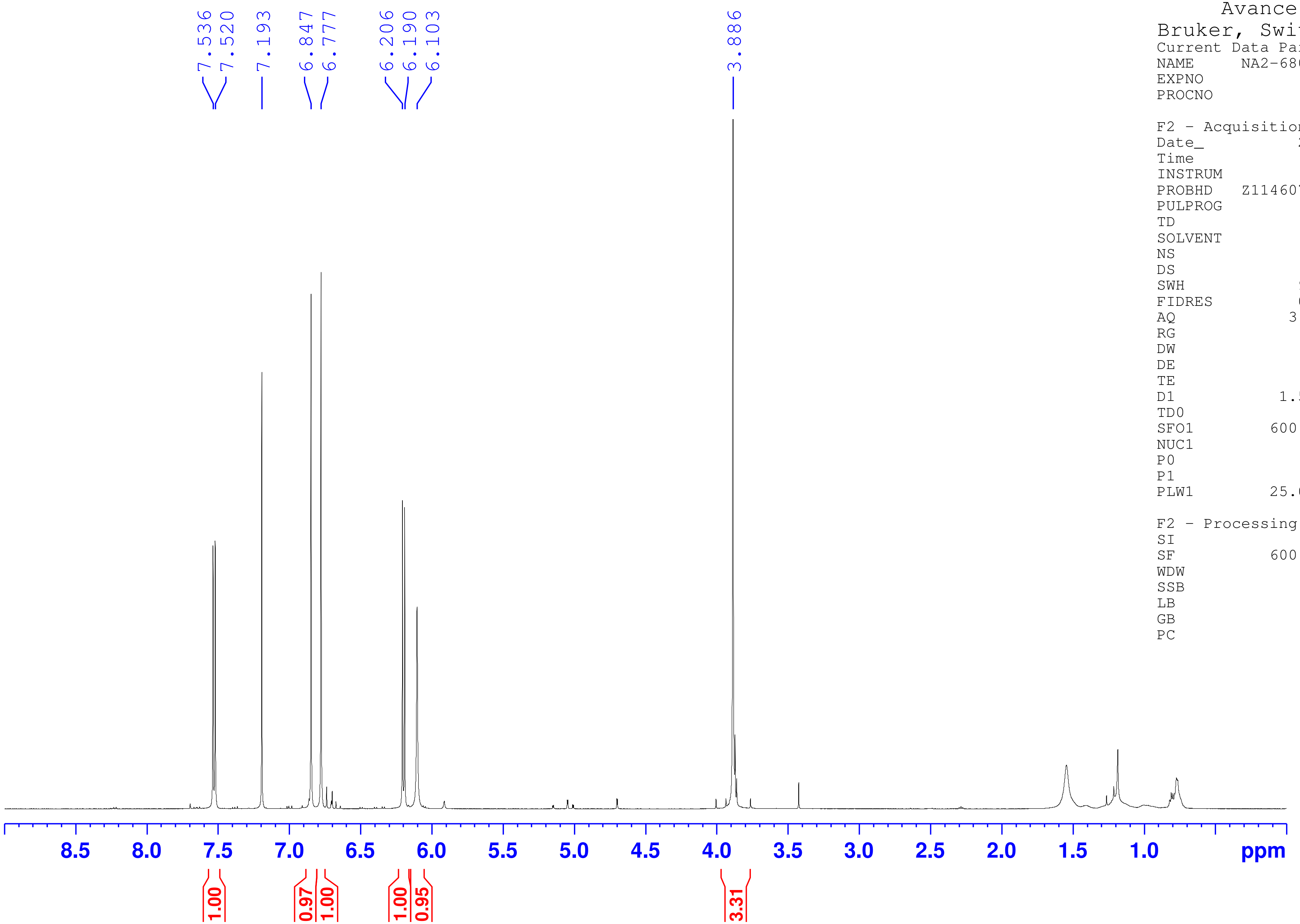

NMR: Ascend TM 600/
Avance III HD
Bruker, Switzerland

Current Data Parameters

NAME	NA2-680838_SC-H-NMR
EXPNO	1
PROCNO	1

F2 - Acquisition Parameters

Date_	20250707
Time	15.38 h
INSTRUM	spect
PROBHD	Z114607_0275 (
PULPROG	zg30
TD	65536
SOLVENT	CDCl3
NS	64
DS	2
SWH	9615.385 Hz
FIDRES	0.293438 Hz
AQ	3.4078720 sec
RG	119.43
DW	52.000 usec
DE	6.50 usec
TE	298.0 K
D1	1.50000000 sec
TD0	1
SFO1	600.1336008 MHz
NUC1	1H
P0	3.33 usec
P1	10.00 usec
PLW1	25.04999924 W

F2 - Processing parameters

SI	65536
SF	600.1300555 MHz
WDW	EM
SSB	0
LB	0.30 Hz
GB	0
PC	1.00

Center of Scientific Equipment for Advanced Research
Thammasat University

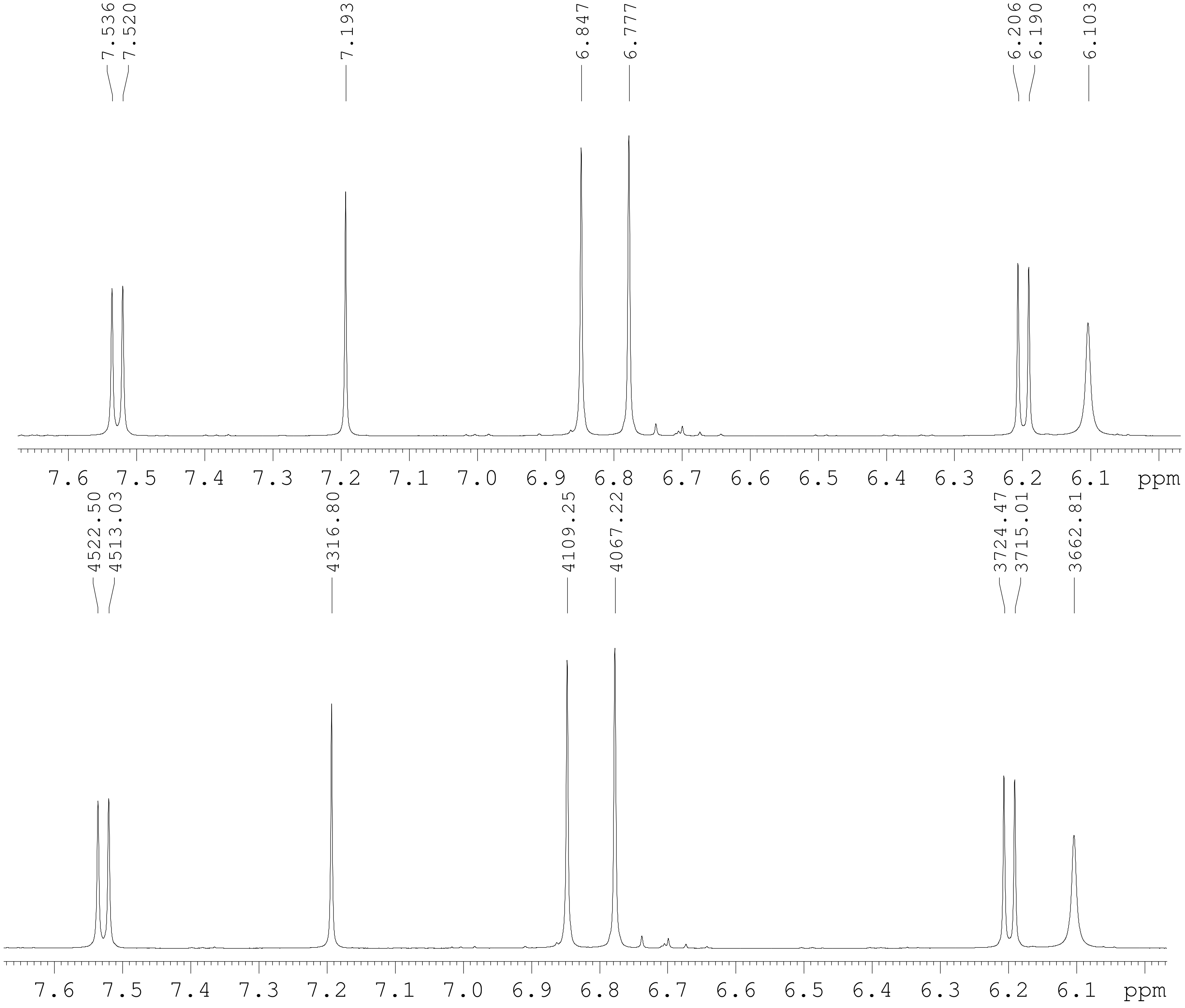

NMR: Ascend TM 600/
Avance III HD
Bruker, Switzerland

Current Data Parameters

NAME	NA2-680838_SC-H-NMR
EXPNO	1
PROCNO	1

F2 - Acquisition Parameters

Date_	20250707
Time	15.38 h
INSTRUM	spect
PROBHD	Z114607_0275 (
PULPROG	zg30
TD	65536
SOLVENT	CDCl3
NS	64
DS	2
SWH	9615.385 Hz
FIDRES	0.293438 Hz
AQ	3.4078720 sec
RG	119.43
DW	52.000 usec
DE	6.50 usec
TE	298.0 K
D1	1.50000000 sec
TD0	1
SFO1	600.1336008 MHz
NUC1	1H
P0	3.33 usec
P1	10.00 usec
PLW1	25.04999924 W

F2 - Processing parameters

SI	65536
SF	600.1300555 MHz
WDW	EM
SSB	0
LB	0.30 Hz
GB	0
PC	1.00
