# Supplementary material for: Natural scopoletin isolated from rubiaceous plants: a precursor for the synthesis of benzoylscopoletin and its cytotoxicity
Source: PeerJ. 2026 May 5;14:e21233. doi: 10.7717/peerj.21233 (PMC13155236; doi:10.7717/peerj.21233)
Supplement: Supplemental Information 2 [file peerj-14-21233-s002.pdf]

Center of Scientific Equipment for Advanced Research
Thammasat University

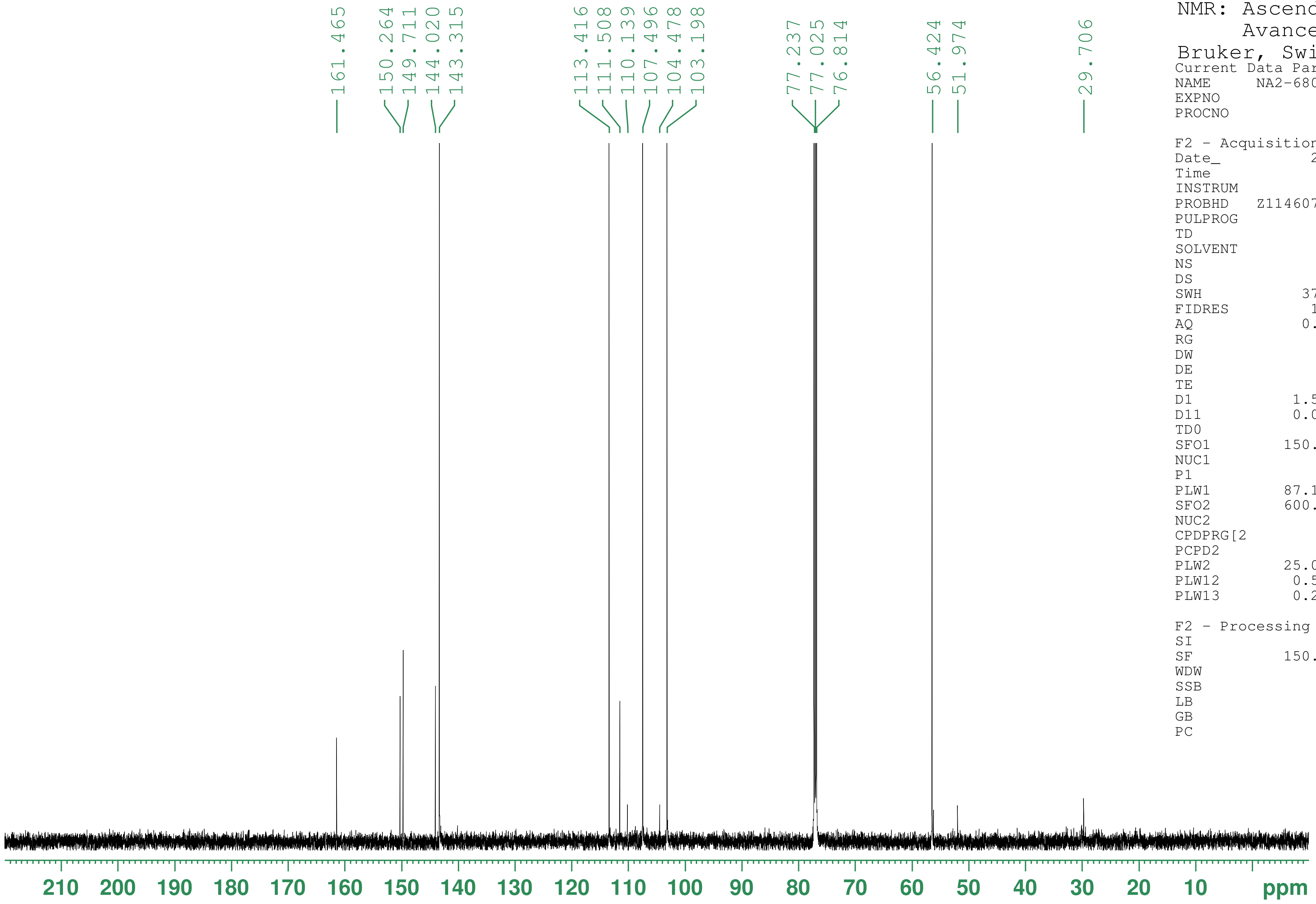

NMR: Ascend TM 600/
Avance III HD
Bruker, Switzerland

Current Data Parameters

NAME	NA2-680838_SC-C-NMR-2
EXPNO	1
PROCNO	1

F2 - Acquisition Parameters

Date_	20250707
Time	18.51 h
INSTRUM	spect
PROBHD	Z114607_0275 (
PULPROG	zgpg
TD	65536
SOLVENT	CDCl3
NS	4500
DS	8
SWH	37878.789 Hz
FIDRES	1.155969 Hz
AQ	0.8650752 sec
RG	191.19
DW	13.200 usec
DE	6.50 usec
TE	298.0 K
D1	1.50000000 sec
D11	0.03000000 sec
TD0	1
SFO1	150.9178988 MHz
NUC1	13C
P1	12.00 usec
PLW1	87.19999695 W
SFO2	600.1324005 MHz
NUC2	1H
CPDPRG[2	waltz16
PCPD2	70.00 usec
PLW2	25.04999924 W
PLW12	0.51121998 W
PLW13	0.25714001 W

F2 - Processing parameters

SI	32768
SF	150.9028085 MHz
WDW	EM
SSB	0
LB	1.00 Hz
GB	0
PC	1.40

Center of Scientific Equipment for Advanced Research
Thammasat University

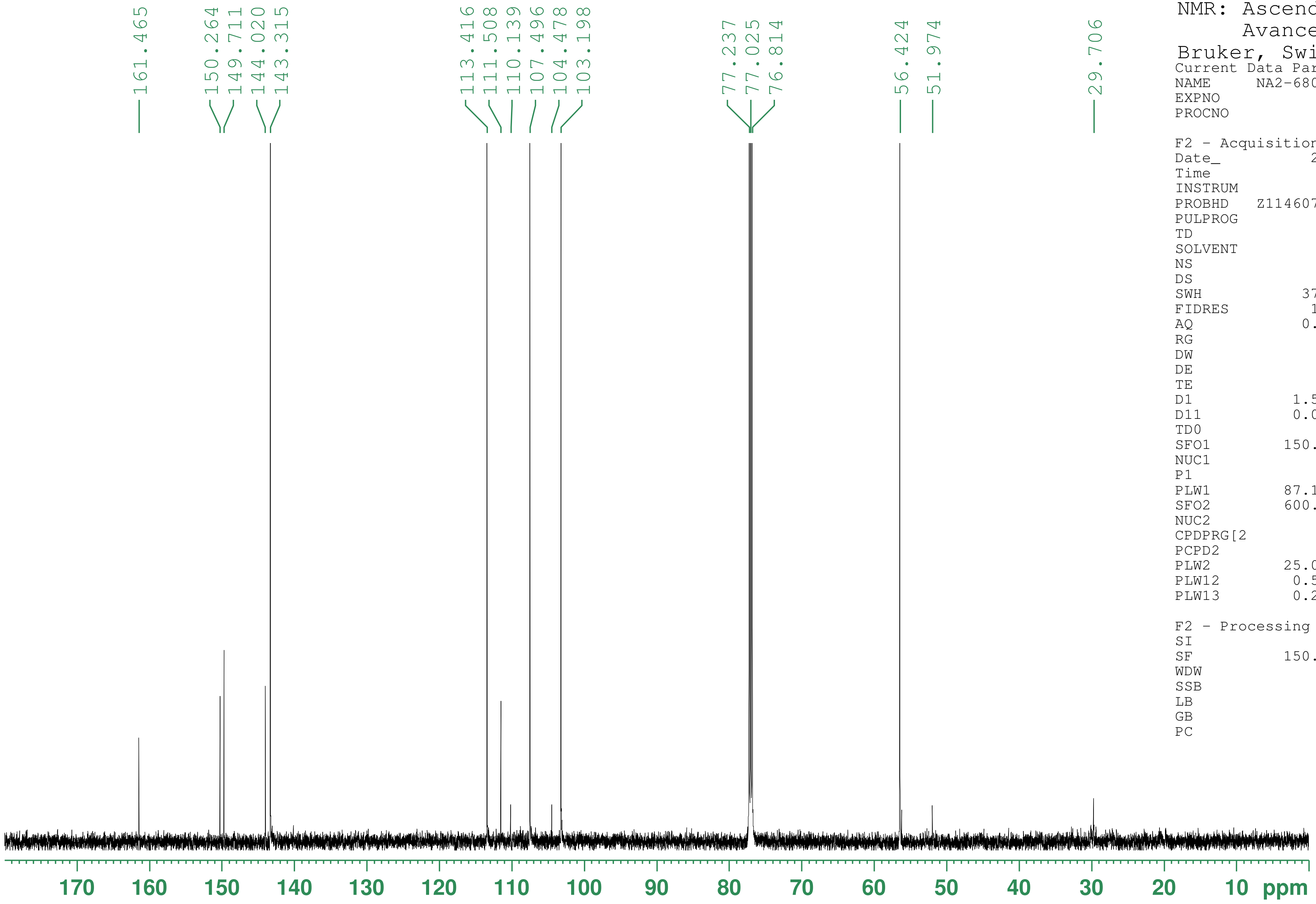

NMR: Ascend TM 600/
Avance III HD
Bruker, Switzerland

Current Data Parameters

NAME	NA2-680838_SC-C-NMR-2
EXPNO	1
PROCNO	1

F2 - Acquisition Parameters

Date_	20250707
Time	18.51 h
INSTRUM	spect
PROBHD	Z114607_0275 (
PULPROG	zgpg
TD	65536
SOLVENT	CDCl3
NS	4500
DS	8
SWH	37878.789 Hz
FIDRES	1.155969 Hz
AQ	0.8650752 sec
RG	191.19
DW	13.200 usec
DE	6.50 usec
TE	298.0 K
D1	1.50000000 sec
D11	0.03000000 sec
TD0	1
SFO1	150.9178988 MHz
NUC1	13C
P1	12.00 usec
PLW1	87.19999695 W
SFO2	600.1324005 MHz
NUC2	1H
CPDPRG[2	waltz16
PCPD2	70.00 usec
PLW2	25.04999924 W
PLW12	0.51121998 W
PLW13	0.25714001 W

F2 - Processing parameters

SI	32768
SF	150.9028085 MHz
WDW	EM
SSB	0
LB	1.00 Hz
GB	0
PC	1.40

Center of Scientific Equipment for Advanced Research
Thammasat University

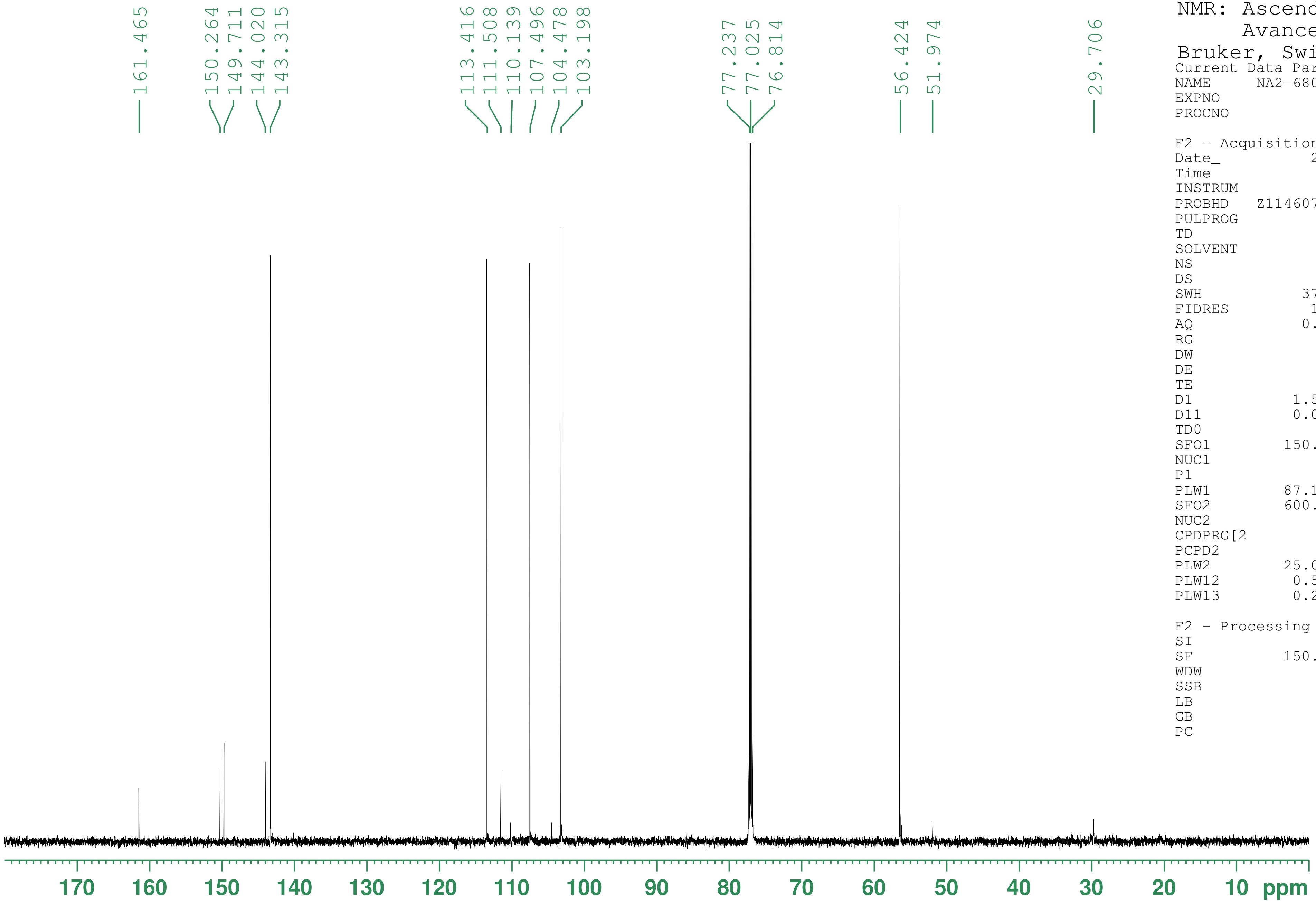

NMR: Ascend TM 600/
Avance III HD
Bruker, Switzerland

Current Data Parameters

NAME	NA2-680838_SC-C-NMR-2
EXPNO	1
PROCNO	1

F2 - Acquisition Parameters

Date_	20250707
Time	18.51 h
INSTRUM	spect
PROBHD	Z114607_0275 (
PULPROG	zgpg
TD	65536
SOLVENT	CDCl3
NS	4500
DS	8
SWH	37878.789 Hz
FIDRES	1.155969 Hz
AQ	0.8650752 sec
RG	191.19
DW	13.200 usec
DE	6.50 usec
TE	298.0 K
D1	1.50000000 sec
D11	0.03000000 sec
TD0	1
SFO1	150.9178988 MHz
NUC1	13C
P1	12.00 usec
PLW1	87.19999695 W
SFO2	600.1324005 MHz
NUC2	1H
CPDPRG[2	waltz16
PCPD2	70.00 usec
PLW2	25.04999924 W
PLW12	0.51121998 W
PLW13	0.25714001 W

F2 - Processing parameters

SI	32768
SF	150.9028085 MHz
WDW	EM
SSB	0
LB	1.00 Hz
GB	0
PC	1.40
