# Supplementary figures and images for: Natural scopoletin isolated from rubiaceous plants: a precursor for the synthesis of benzoylscopoletin and its cytotoxicity
Source: PeerJ. 2026 May 5;14:e21233. doi: 10.7717/peerj.21233 (PMC13155236; doi:10.7717/peerj.21233)

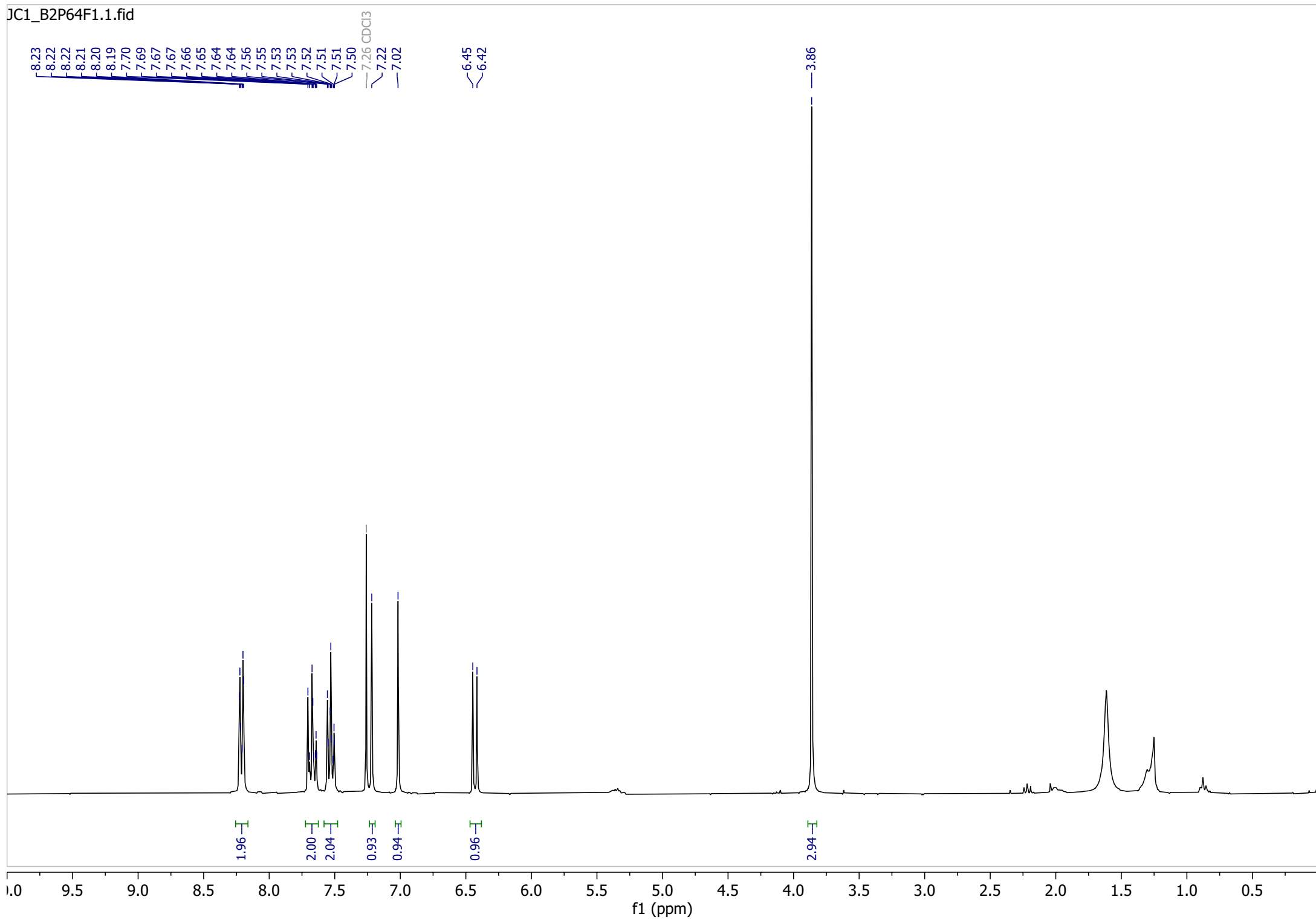

Supplement: Supplemental Information 3 [file peerj-14-21233-s003.pdf]

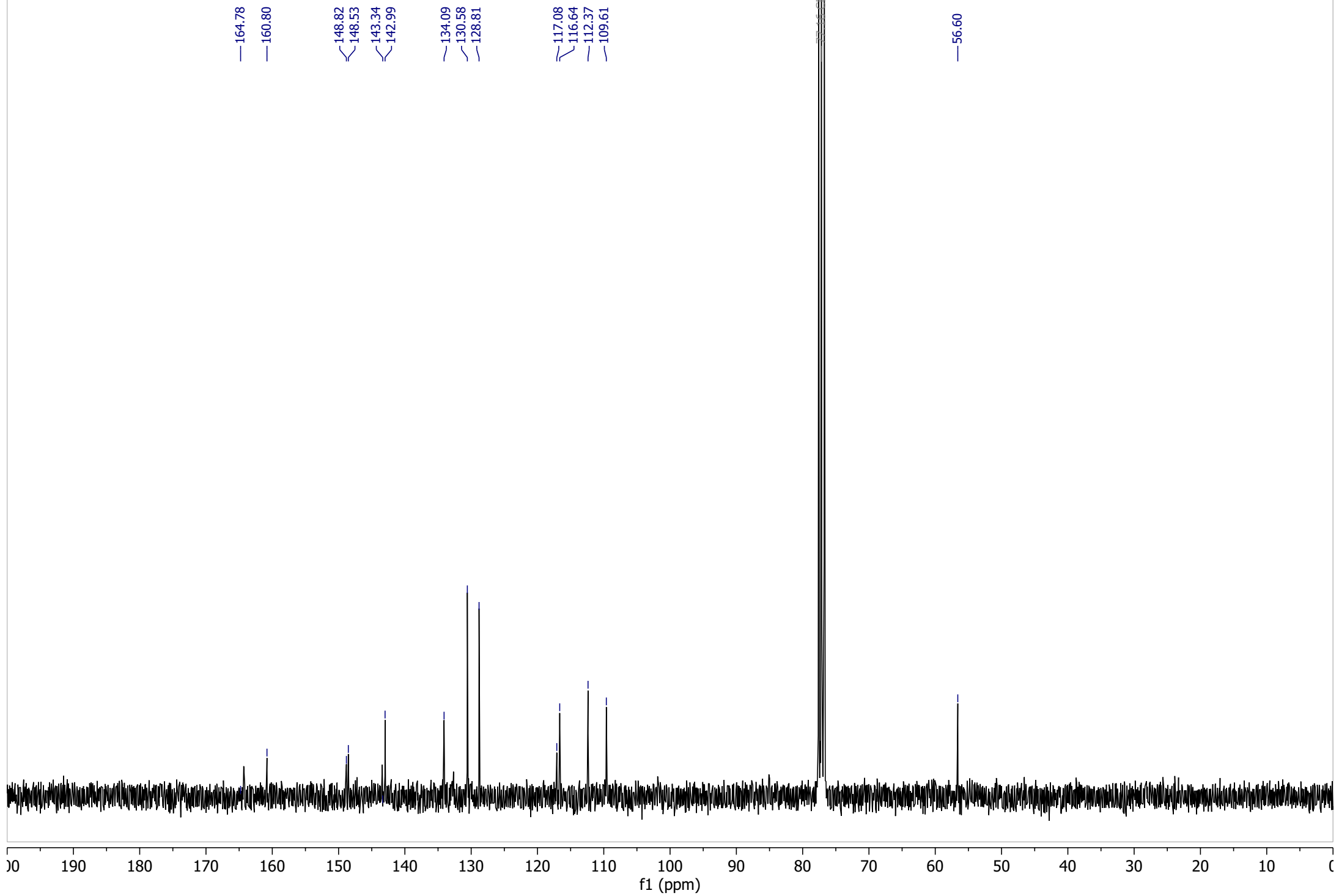

Supplement: Supplemental Information 4 [file peerj-14-21233-s004.pdf]
